# eIF3d: A driver of noncanonical cap–dependent translation of specific mRNAs and a trigger of biological/pathological processes

**DOI:** 10.1016/j.jbc.2023.104658

**Published:** 2023-03-29

**Authors:** Shijie Ma, Jing-Yuan Liu, Jian-Ting Zhang

**Affiliations:** 1 Department of Cell and Cancer Biology, The University of Toledo College of Medicine and Life Sciences, Toledo, Ohio, USA; 2 Department of Medicine, The University of Toledo College of Medicine and Life Sciences, Toledo, Ohio, USA

**Keywords:** eIF3d, cap-dependent translation, tumorigenesis, drug resistance, signaling pathway

## Abstract

Eukaryotic initiation factor 3d (eIF3d), a known RNA-binding subunit of the eIF3 complex, is a 66 to 68-kDa protein with an RNA-binding motif and a cap-binding domain. Compared with other eIF3 subunits, eIF3d is relatively understudied. However, recent progress in studying eIF3d has revealed a number of intriguing findings on its role in maintaining eIF3 complex integrity, global protein synthesis, and in biological and pathological processes. It has also been reported that eIF3d has noncanonical functions in regulating translation of a subset of mRNAs by binding to 5′-UTRs or interacting with other proteins independent of the eIF3 complex and additional functions in regulating protein stability. The noncanonical regulation of mRNA translation or protein stability may contribute to the role of eIF3d in biological processes such as metabolic stress adaptation and in disease onset and progression including severe acute respiratory syndrome coronavirus 2 infection, tumorigenesis, and acquired immune deficiency syndrome. In this review, we critically evaluate the recent studies on these aspects of eIF3d and assess prospects in understanding the function of eIF3d in regulating protein synthesis and in biological and pathological processes.

Translational control is a fundamental regulatory mechanism of gene expression. Abnormal translational control has been associated with human diseases such as cancer (1, 2, 3, 4). Translation of mRNAs is mainly regulated at initiation, the rate-limiting step (5, 6) that requires at least 12 eukaryotic initiation factor (eIF) complexes (7, 8, 9). In eukaryotes, the canonical cap–dependent translation initiation is a complicated process involving formation of several different complexes of eIFs, ribosomes, mRNAs, and tRNAs as well as scanning of 5′-UTRs in mRNAs (5). Briefly, the Met-tRNAi-eIF2–GTP tertiary complex is recruited to the 40S ribosome complexed with eIF1, eIF3, and eIF5, forming the 43S preinitiation complex (PIC). Meanwhile, capped mRNA molecules bind to eIF4B and eIF4F (consisting of eIF4A, 4E, and 4G) at their 5′-end cap structures, which help recruit the 43S PIC, leading to the formation of the 48S PIC. The 48S PIC then scans the 5′-UTR, searching for the translation initiation start codon. With release of eIFs at the start codon, the 60S ribosome joins the 40S ribosome to form the 80S ribosome, which begins translation of the mRNA. In this canonical cap–dependent translation initiation process, the eIF3 complex is thought to promote translation initiation by binding to the 40S ribosome to keep it dissociated from the 60S ribosome, to stabilize the binding of the eIF2•GTP•Met–tRNAi tertiary complex to the 40S ribosome, and to promote maximal binding of the eIF4F–mRNA complex to the 40S ribosome in forming PICs (10).

Among all eIFs, eIF3 is the largest multisubunit complex (11, 12) with variable compositions in different species (13, 14, 15). While human eIF3 consists of 13 subunits designated eIF3a–eIF3m (Fig. 1), the eIF3 in *Saccharomyces cerevisiae* consists of only six subunits including eIF3a, b, c, g, i, and j. The eIF3 in *Schizosaccharomyces pombe* is more complex than that in *S. cerevisiae* with 11 subunits including eIF3a, b, c, d, e, f, g, h, i, j, and m. Interestingly, the *Neurospora crassa* eIF3 has all 13 subunits, same as that of the human eIF3. The observation of different compositions in the eIF3 complex of different species raises an interesting question on the necessity of all 13 subunits for eIF3 function in translation initiation and if some subunits may play only structural or regulatory function in translating specific mRNAs.

**Figure 1:**
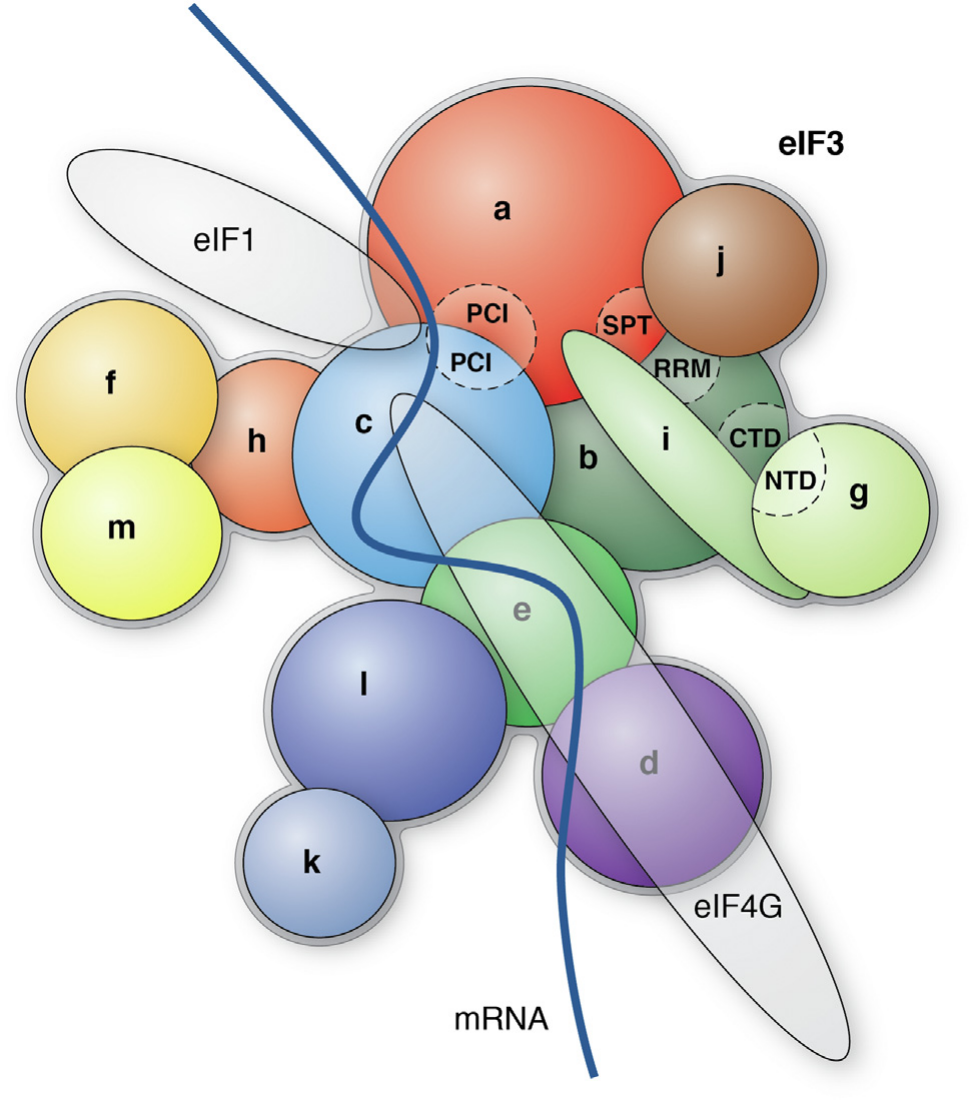
**Schematic model of human eIF3 complex and its binding partners with specific motif indicated for protein–protein interactions** (31, 33, 36, 39, 94, 95, 96, 97, 98, 99)**.** This model is adapted from Susan *et al.* (36) with modification. CTD, carboxyl terminal domain; eIF3, eukaryotic initiation factor 3; PCI, proteasome–COP9 complex–initiation; RRM, RNA recognition motif; SPT, spectrin.

In addition to participation in nearly every step of the canonical cap–dependent translation initiation as described previously, eIF3 may also be involved in controlling other processes of protein synthesis, including elongation, termination, and quality control in both positive and negative modes (12). Furthermore, eIF3 subunits may also have multiple specific noncanonical functions to activate or repress translation of different subgroups of mRNAs independent of the eIF3 complex (13, 16, 17, 18). In this regard, eIF3d is of particular interest and likely drives translation initiation of a select group of mRNAs in a cap-dependent and eIF4F-independent manner. There is also evidence suggesting that eIF3d may regulate protein stability in addition to protein synthesis, which together trigger biological and pathological processes.

In this review, we focus on recent progress in investigating the role of eIF3d in the eIF3 complex formation, translational regulation, metabolic stress adaptation, and pathological processes. Although it has been shown that eIF3d is required for the eIF3 complex formation in fission yeast (19), other studies showed that eIF3d might be dispensable and not required for the stable eIF3 complex integrity in human cells (20). A number of additional studies have also shown that eIF3d is involved in tumorigenesis *via* translational regulation of oncogene expression (18, 21, 22, 23, 24), suggesting that it may serve potentially as a novel biomarker and cancer therapeutic target. Moreover, eIF3d may play an important role in infectious diseases and assist infection by viruses such as severe acute respiratory syndrome coronavirus 2 (SARS-CoV-2) (25).

## Molecular structure of eIF3d

**Figure 2:**
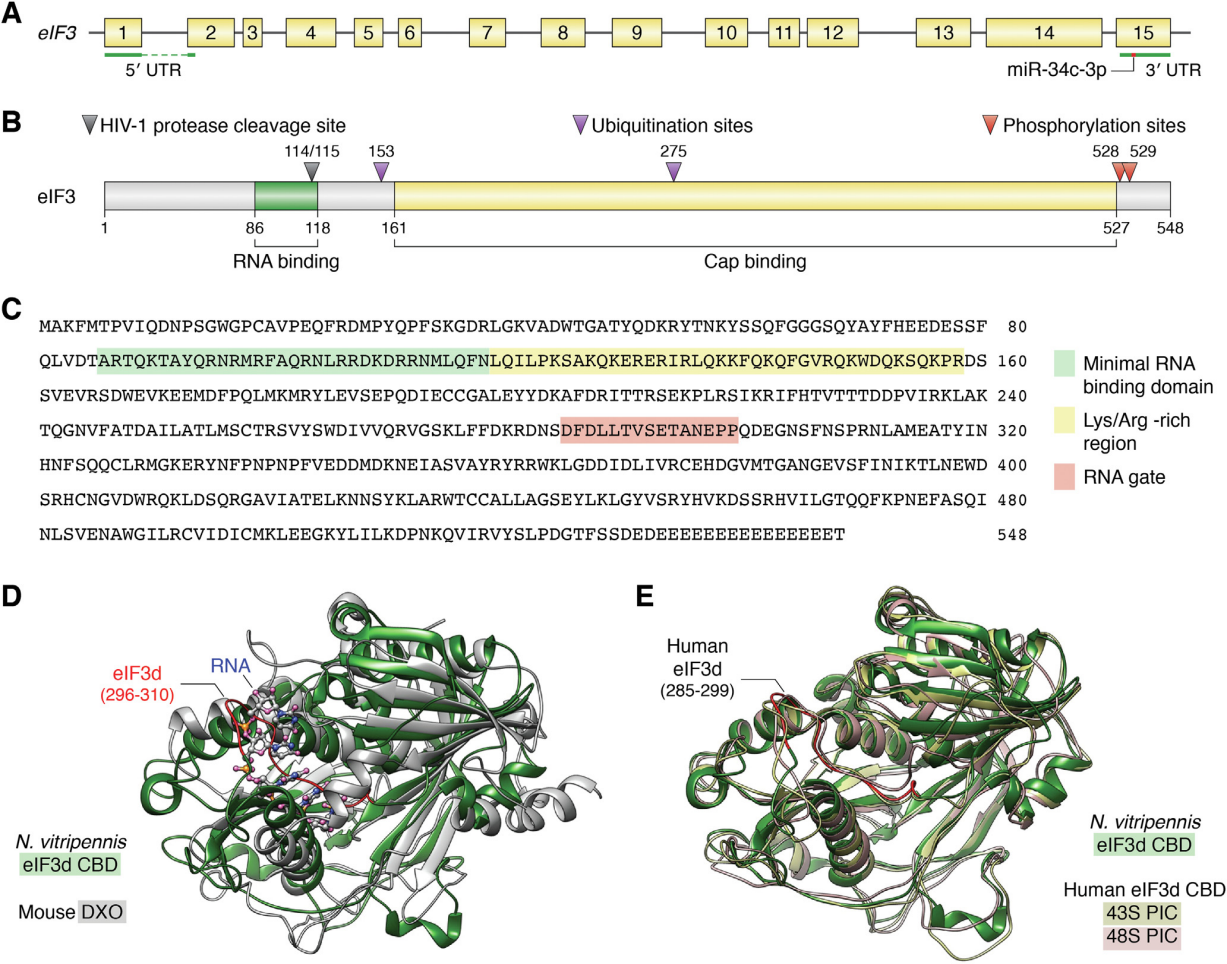
**Genomic structure and protein domains of human eIF3d.***A*, linear scheme of human eIF3d genome structure. Exons are shown as *numbered yellow boxes* with the exons encoding UTRs indicated by *green-underlinedboxes*. The binding site for miR-34c-3p in the 3′-UTR region is indicated. *B*, schematic linear structure of eIF3d protein with RNA-binding domain and cap-binding domain indicated in *green* and *yellow*, respectively, and amino acid residue positions labeled. The sites of HIV-1 protease cleavage, ubiquitination, and phosphorylation are indicated. *C*, amino acid sequence of human eIF3d protein with different domains and sequences indicated. *D*, the structural alignment of *Neurospora vitripennis* eIF3d CBD (*green*, Protein Data Bank [PDB] code: 5K4B) and murine DXO (in *gray*, PDB code: 4J7L) with a root mean square deviation of 1.29 Å. The single-stranded RNA complexed with DXO is shown by ball-and-stick representation. Residues 296 to 310 in *N. vitripennis* eIF3d CBD that occupies the mRNA channel and clashes with the single-strand RNA in DXO is colored in *red*. *E*, structural alignment of human eIF3d CBD in the open state 43S PIC (*gold*, PDB code: 7A09) and in the open state 48S PIC (*peach*, PDB code: 7QP6) with *N. vitripennis* eIF3d CBD (*green*, positioned same as in *C*). In both 43S and 48S PIC, residues 285 to 299 of human eIF3d align with residues 296 to 310 of *N. vitripennis* eIF3d CBD and equally occupy the mRNA channel. CBD, cap-binding domain; DXO, decapping and exoribonuclease; eIF3, eukaryotic initiation factor 3; PIC, preinitiation complex.

eIF3d, also known as p66, EIF3S7, eIF3-zeta, and eIF3-p66 in human, *Drosophila*, and Moe1 in fission yeast *S. pombe*, is a 66-kDa protein consisting of 548 amino acid residues in human and a 68-kDa protein of 567 amino acids in fission yeast (26, 27). Mammalian elF3d was first identified as a major RNA-binding subunit of the eIF3 complex purified from rabbit reticulocyte lysate (28). *eIF3d* is highly conserved among different higher species including *Drosophila* (29). Human *eIF3d* gene has been mapped to chromosome 22q13.1 with 15 exons of 46 to 284 bases each, spanning approximately 18 kb (Fig. 2*A*). Comparison between the *eIF3d* sequence and sequences in the expressed sequence tag database indicates that it is abundantly and ubiquitously expressed (29).

Human, *Drosophila*, *N. crassa*, and *S. pombe* eIF3d complementary DNAs have been cloned (14, 26, 29, 30), and the amino acid sequence contains an RNA-binding domain and a cap-binding domain (CBD) (Fig. 2*B*) (18, 29). The minimal RNA-binding domain in human eIF3d was mapped to the amino terminal end consisting of amino acid residues 86 to 118 using recombinant glutathione-*S*-transferase-eIF3d fusion proteins with sequential deletions in combination with Northwestern analysis of RNA-binding activities of these proteins (29). It was also thought that the RNA-binding domain could extend to amino acid residue 158 including a lysine/arginine-rich region (Fig. 2*C*).

The CBD of eIF3d is between residues 161 and 527 (18). Using sulfur anomalous dispersion for phase determination, a 1.4 Å crystal structure of the eIF3d CBD from *Neurospora vitripennis* with 65% identical sequence to that of human eIF3d was determined. Surprisingly, although sequence similarity is low, the eIF3d CBD of *N. vitripennis* shares the same fold as that of murine decapping and exoribonuclease protein (18), which has pyrophosphohydrolase activity and can decap and degrade incompletely-capped pre-mRNAs and is involved in pre-mRNA 5′-end capping quality control. This observation along with other evidence suggests that eIF3d may play a role in regulating cap recognition for translation initiation of specific mRNAs such as *c-Jun* mRNA independent of eIF4F (18). However, structural alignment of the *N. vitripennis* eIF3d CBD domain with murine decapping and exoribonuclease–mRNA complex shows that a 15-amino-acid insertion comprising residues 296 to 310 (equivalent to residues 285–299 in human, Fig. 2*C*) occupies the mRNA-binding channel (Fig. 2*D*). This insertion is subsequently termed as “RNA gate” as it takes the space and prohibits mRNA binding. It is hypothesized that eIF3d must undergo a conformational change to release this insertion from the channel to receive an mRNA it regulates. The existence of this putative RNA gate in human eIF3d has been confirmed by other studies (31, 32) However, an “open gate” eIF3d or eIF3d complexed with an mRNA 5′-cap or a single-strand RNA in the channel has not yet been captured. Structural alignment of human eIF3d CBD in the 43S and 48S PICs with *N. vitripennis* eIF3d CBD shows that the putative gate of eIF3d in the PICs equally occupies the mRNA channel in a closed conformation (Fig. 2*E*), This lack of observation of the “open gate” conformation suggests that eIF3d may be strictly regulated and other factors, such as death-associated protein 5 (DAP5, see discussion later), are possibly required for this conformational transition and subsequent mRNA recognition.

In the structure of the mammalian 48S PIC containing all 13 eIF3 subunits obtained using single-particle cryo-EM, eIF3 appears to interact with the 40S ribosome, 18S rRNA, and ribosomal proteins *via* different eIF3 subunits (31, 33). Particularly, eIF3d contributes to the interaction between the eIF3 complex and the 40S ribosome *via* interacting with eIF3a, c, and e in eIF3 and the 40S ribosome (33). Moreover, the N-terminal tail of eIF3d binds to the proteasome–COP9–initiation factor domain of eIF3c *via* its conserved Trp^45^ and the Pro^603^, Gln^606^, Ile^607^, and Glu^666^ residues in eIF3c (31, 33). While these structures provide important information in understanding the interactions between these proteins and rRNAs and different conformational state of the PICs, little is known regarding the direct function of eIF3d in these interactions. It is also unclear how the position of eIF3d domains coordinate the vital functions during the canonical translation initiation. Structural data with eIF3d in action would be desirable. Approaches including the use of Bio-small-angle X-ray scattering (Bio-SAXS) to capture solution structures of eIF3d in action or its dynamic conformation with or without other eIF3 subunits, mRNAs, and ribosomes may help delineate these issues. However, the structural data should also be approached with caution because the isolated proteins or complexes are studied outside living cells and are enriched in high concentrations with reconstitution, which could create interactions that may not exist in living cells.

## Biological functions of eIF3d

Although eIF3d is a subunit of the eIF3 complex in some species (30) and it may play an important role in the 48S PIC formation (see aforementioned), the fact that it does not exist in the eIF3 complex in *S. cerevisiae* is very intriguing. Indeed, it was later found that eIF3d might be dispensable in forming the stable eIF3 complex and in global translation initiation (20, 30). eIF3d may also have unique functions as an independent regulator in controlling translation of a specific group of mRNAs and in regulating protein stability. More recently, accumulating evidence suggests that eIF3d may have other regulatory functions in metabolic stress adaptation.

### Translational regulation

The finding that eIF3d regulates translation initiation was first reported in *S. pombe* with eIF3d deletion causing moderate inhibition (30–40%) in global protein synthesis (19). Based on this finding, it was thought that eIF3d may play a role in optimizing global translation initiation or regulating translation of a specific subset of mRNAs, which contributes to a partial inhibition of global protein synthesis following its deletion. It is noteworthy, however, that the eIF3 complex may have lost its integrity after eIF3d deletion since other eIF3 subunits could not be coimmunoprecipitated with eIF3e in the eIF3d deletion mutant strain (19). This finding was confirmed by characterization of eIF3 subunits associated with the 40S ribosome, which also indicated that the interaction among eIF3 subunits was lost with eIF3d deletion (19). Thus, eIF3d may be important in maintaining the integrity of the eIF3 complex in *S. pombe*. However, it is unclear why the disruption of the eIF3 complex integrity because of eIF3d deletion resulted in only 30 to 40% inhibition in global protein synthesis. Although it is possible that eIF3d may regulate translation of a subset of mRNAs that contribute to partial inhibition of global protein synthesis after the loss of eIF3 complex integrity because of eIF3d deletion, eIF3d would have to suppress translation of a large subset of mRNAs. Alternatively, the eIF3 complex without eIF3d may still exist and partially functional but much less stable, which could account for its failure to be isolated using coimmunoprecipitation.

In contrast to the findings in fission yeast, eIF3d downregulation using siRNA in human HeLa cells produced severe defects in global protein synthesis with less than 10% remaining compared with control cells (20). This finding suggests that eIF3d may be required for global translation initiation in mammalian cells. In subsequent studies, however, it was found that *eIF3d* knockdown had effect on neither the protein levels of other eIF3 subunits nor the integrity of the eIF3 complex, similar to the finding with eIF3j (20). Thus, it is unclear how *eIF3d* knockdown essentially eliminated global protein synthesis without affecting the integrity of the eIF3 complex although it is possible that eIF3d is required for eIF3 function in global translation initiation but not required in eIF3 complex assembly in HeLa cells.

Previously, it was widely accepted that the eIF3 complex is required in global translation initiation (34, 35). The studies on eIF3d and the integrity of eIF3 complex in two different model systems result in challenges to this dogma. Nevertheless, in contrast to other subunits such as eIF3b, i, g, and c, which are required for the stable eIF3 complex formation (20, 36), eIF3d may be dispensable in human cells although it may be required for the eIF3 complex integrity in fission yeast. Domain mapping and/or structural studies of the eIF3 complex as discussed previously may help elucidate how eIF3d functions in maintaining the eIF3 complex integrity and in global protein synthesis in both fission yeast and human cells. It is also necessary to determine if eIF3d in maintaining eIF3 complex integrity is independent from its role in supporting eIF3 function in global translation initiation in different species.

**Figure 3:**
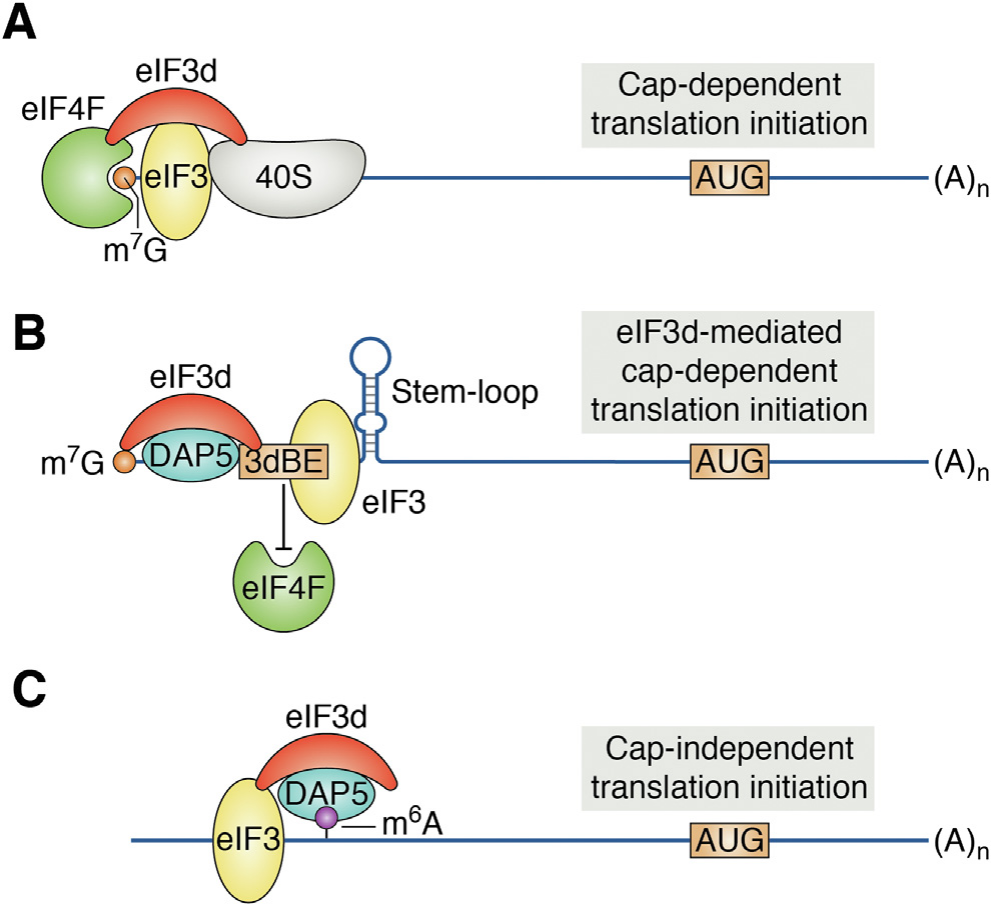
**Schematic models of canonical and noncanonical translation initiations**. *A,* eIF4F-mediated canonical cap–dependent translation initiation. *B* and *C*, eIF3d-mediated non–canonical cap–dependent (*B*) and can–independent (*C*) translation initiation that are independent of eIF4F. 3dBE, eIF3d-binding elements in the 5’-UTR; eIF, eukaryotic initiation factor.

eIF3d has also been reported to interact directly with the 5′-cap in mRNAs (18), indicating that eIF3d may help recruit mRNAs to the 43S PICs independent of eIF4F. Thus, there may be an alternate or noncanonical cap–dependent initiation mechanism *via* eIF3d, different from the canonical cap–dependent translation initiation *via* eIF4F (Fig. 3, *A* and *B*). However, it is unclear if this function of eIF3d requires the eIF3 complex in the noncanonical cap–dependent translation initiation of these mRNAs.

Using transcriptome-wide identification by photoactivatable ribonucleoside-enhanced crosslinking and immunoprecipitation, it was demonstrated that the eIF3 complex is necessary for the translation of a specific subset of mRNAs by directly binding to stem–loop structures in their 5′-UTRs (10, 16, 18, 23). Functional studies of the interactions were conducted further for two mRNAs encoding the cell proliferation regulators *c-Jun* and B-cell translocation gene 1 (BTG1), which uncovered that eIF3 mediates translation activation or repression using different types of RNA stem–loop binding (16, 18). Furthermore, separation of the crosslinked eIF3–RNA complexes using denaturing gel electrophoresis followed by mass spectrometry analyses revealed four eIF3 subunits, eIF3a, b, d, and g, that were RNA bound. To determine whether all four eIF3 submits might be involved in 5′-cap recognition for efficient translation, the authors crosslinked the purified eIF3a, b, d, and g to ^32^P-internal or ^32^P-cap-labeled *c-Jun* 5′-UTR RNA and resolved them by SDS-PAGE gel shift (18). Phosphorimage of SDS-PAGE showed that only the purified eIF3d could bind to the 5′-cap of *c-Jun* mRNA, and the identification of eIF3d was further verified by limited proteolysis and mass spectrometry. This finding suggests that eIF3d may participate in a specialized mechanism of translation initiation for a subset of mRNAs possibly *via* binding to the 5′-cap in these mRNAs. This possibility is supported by the fact that eIF3d is located in the mRNA exit channel in the eIF3 complex structure and may regulate cap recognition as discussed previously with assumption that the eIF3 complex is required for this noncanonical cap–dependent translation initiation.

While the aforementioned possibility is very provocative, it is noteworthy that the structure showing eIF3d binding to or interacting with the m^7^G cap has yet to be resolved. The lack of this evidence weakens but does not rule out this possibility since X-ray diffraction or cryo-EM requires ordered structure to be detected. Binding of the m^7^G cap to eIF3d may cause disorder or increased flexibility of the protein, resulting in lost crystal packing for structural determination, although this may not occur with other cap-binding proteins. Use of other approaches to determine solution structure such as Bio-SAXS as discussed previously may be helpful to provide the needed structural information with the use of m^7^G-capped mRNA.

Interestingly, eIF3l has also been shown to bind to the m^7^G cap (37), suggesting that eIF3l–cap interaction may also play a role in the translation of specific mRNAs with m^7^G caps that have high affinity to eIF3l. However, structural evidence on this interaction is also lacking. While the findings that different eIF3 subunits may bind to m^7^G caps to initiate noncanonical cap–dependent translation are intriguing, eIF3d and eIF3l may do so for different mRNAs. It is of interest in future studies to determine if eIF3d and eIF3l are mutually exclusive and compete for binding to specific mRNAs or each has its own select group of mRNAs with m^7^G caps using *in vitro* RNA-binding or pull-down assays of purified recombinant proteins. It is also necessary to determine how eIF3d and eIF3l help initiate cap-dependent translation by coordinating with other factors and proteins including ribosomes.

Using luciferase reporter assay of the *c-Jun* 5′-UTR with or without the internal eIF3-recruitment stem–loop sequence (nucleotide position 181–214) (16), it was found that an RNA element (nucleotide positions 67–153) in the 5′-UTR of *c-Jun* mRNA directed the mRNA to use the hypothetical eIF3d-mediated translation initiation (18). Using Western blot analysis of initiation factors in the 48S PIC formed on *c-Jun* mRNA, it was found that the full-length mRNA was unable to recruit eIF4F components (eIF4G1, eIF4A1, and eIF4E). However, the 48S PIC formed on *c-Jun* mRNA lacking the first 153 nucleotides in the 5′-UTR could recruit eIF4F factors. These results indicate that the sequence in the 5′-UTR of *c-Jun* mRNA (nucleotide position 67–153) important for eIF3d recruitment may be inhibitory to eIF4F binding because of eIF3 binding or secondary structure of the sequence. These possibilities could be further tested by identifying other mRNAs that may associate with eIF3d but not eIF4F to determine whether there is a consensus sequence or elements for eIF3d binding and the mechanism of excluding eIF4F (see discussion later).

It is noteworthy that in canonical cap–dependent translation initiation, the slotting mechanism by which mRNA binds to eIF4F and the 40S ribosome leaves a 30 to 40 nucleotide “blind spot” toward the 5′-end in the 5′-UTR of mRNAs and therefore favors translation at the start codon more than 50 bases from the 5′-end (33). This observation suggests that eIF3d binding in the position 67 to 153 in the 5′-UTR of *c-Jun* mRNA may sterically hinder eIF4F binding to the 5′-end of the 5′-UTR. Deletion of the eIF3d-binding sequence should effectively remove the eIF3d inhibition of eIF4F binding. Based on these observations, it appears that *c-Jun* mRNA may represent a noncanonical mRNA that requires eIF3d for its translation and does not use the eIF4F-dependent canonical pathway. Clearly, more studies such as eIF4E knockdown effect on *c-Jun* expression are needed to eliminate the possibility that it uses the canonical cap–dependent initiation and to determine what other mRNAs behave similarly as *c-Jun* mRNA. Indeed, it was found recently that eIF3d is required for translation initiation of the mRNA encoding the core U2 spliceosomal component protein SF3A3 during Myc-driven oncogenesis and that eIF3d function is dependent on a stem–loop structure in the 5′-UTR of *SF3A3* mRNA (23), similar to that in the 5′-UTR of *c-Jun* mRNA (Fig. 3*B*). Thus, there is evidence that a subpopulation of mRNAs exist that do not use the canonical cap–dependent mechanism but rather an eIF3d-dependent noncanonical pathway for their translations.

Consistent with the findings discussed previously, eIF3d also promotes translation of capped mRNAs encoding matrix metalloproteinase 1 and cyclin-dependent kinase 12 (CDK12) independent of eIF4F complex but by interacting with DAP5 (Fig. 3*B*), a member of the eIF4G family that includes DAP5 (also called eIF4G2), eIF4GI (eIF4G1), and eIF4GII (eIF4G3) (24, 38). Unlike eIF4GI/II, DAP5 lacks the eIF4E-binding domain and, indeed, DAP5 does not bind to eIF4E as shown using coimmunoprecipitation (24). Thus, DAP5 may participate in eIF3d-mediated noncanonical cap–dependent translation initiation but not in canonical cap–dependent initiation. Since eIF3d has also been shown to participate in eIF3 binding to eIF4GI/II in eIF4F to promote the binding of the 43S PIC to mRNA in the canonical translation initiation (39), eIF3d likely has dual roles in translation initiation. However, it is unknown if the binding of DAP5 and eIF4GI/II to eIF3d is mutually exclusive at the same site on eIF3d and if the eIF3d–DAP5 interaction in noncanonical cap–dependent initiation requires other eIF3 subunits and how it interacts with ribosomes. Nevertheless, it has been shown using stringent coimmunoprecipitation that eIF3d strongly binds to DAP5 but weakly to eIF4GI/II (24). While this finding suggests that eIF3d prefers to bind to DAP5 over eIF4GI/II to exercise its noncanonical function, it has been shown that the interaction of the eIF3 complex with eIF4GI/II also involves eIF3c and eIF3e, which together likely strengthen the interaction between eIF3 and eIF4F complexes in canonical cap–dependent initiation.

It is noteworthy, however, that the eIF3d–DAP5 complex–mediated noncanonical cap–dependent mRNA translation generally occurs with mammalian target of rapamycin (mTOR) inhibition and eIF4E depletion. In a recent study of translational regulation in human CD4+ T-cell differentiation, it was found that a set of noncanonical mRNAs important for the development of regulatory T cells (Treg) used the eIF3d/DAP5-dependent noncanonical mechanism for their translation when mTOR is inhibited and the eIF4E-cap–dependent translation is impaired (38). While the mechanism of eIF3d/DAP5-driven translation remains to be solved, their binding to the 5′-UTR and to the m^7^G cap but with less competitive activity than eIF4E binding to the cap (38) suggests that other structural or sequence motifs may exist in the 5′-UTR that facilitate eIF3d/DAP5-mediated translation initiation. Although no such consensus sequences or secondary structures have been identified in these mRNAs except GC-rich motifs, it is still possible that stem–loop secondary structures exist in the 5′-UTR of these mRNAs (see discussion previously on *c-Jun* and *SF3A3* mRNAs). These possibilities should be tested in future studies to understand the detailed molecular mechanism of eIF3d/DAP5 regulation of mRNA translation.

eIF3d and DAP5 may also coordinate in the noncanonical cap–independent translation initiation *via* binding to N^6^-methyladenosine (m^6^A) in the 5′-UTR of mRNAs. In this regard, DAP5 has been reported to promote circular RNA (circRNAs) translation *via* recognizing m^6^A modification (40). Using m^6^A immunoprecipitation and RT–PCR, it was found that m^6^A and DAP5-binding sites were relatively enriched in circRNAs and m^6^A is located upstream of the DAP5-binding sites, suggesting that DAP5 may functionally cooperate with m^6^A in driving circRNA translation by a cap-independent fashion (40). Interestingly, eIF3d has been shown to bind to m^6^A-containing RNAs (41). It is thus possible that eIF3d and DAP5 coordinate in the noncanonical cap–independent translation initiation *via* binding to m^6^A (Fig. 3*C*). However, information on this novel mechanism of cap-independent translation initiation is limited, and more in-depth study is needed to determine the molecular basis of eIF3d and DAP5 involvement and coordination as well as other factors contributing to this process.

It has also been reported that eIF3d interacts with the RNA-binding protein RNA binding motif single stranded interacting protein 1 (RBMS1) and mediates RBMS1 activation of solute carrier family 7 member 11 (SLC7A11) mRNA translation (22). It was shown that RBMS1 directly binds to eIF3d using pull-down assay of purified proteins and that both RBMS1 and eIF3d are required for SLC7A11 expression. It was thought that RBMS1 bridges the 3′- and 5′-UTR of *SLC7A11* mRNA by binding to eIF3d to activate the translation of *SLC7A11* mRNA (22). While these findings suggest that eIF3d may have a noncanonical function in driving translation of *SLC7A11* mRNA by interacting with RBMS1, it is unclear if other eIF3 subunits and/or eIF4F are required for synthesis of SLC7A11 proteins. Indeed, eIF3i and eIF3m were also coimmunoprecipitated with RBMS1 although it appears that they were indirectly coprecipitated *via* eIF3d. Nevertheless, based on these and the aforementioned findings, the involvement of eIF3d in regulating translation of specific mRNAs is more complex than our current level of understanding of regulation in translation initiation.

**Figure 4:**
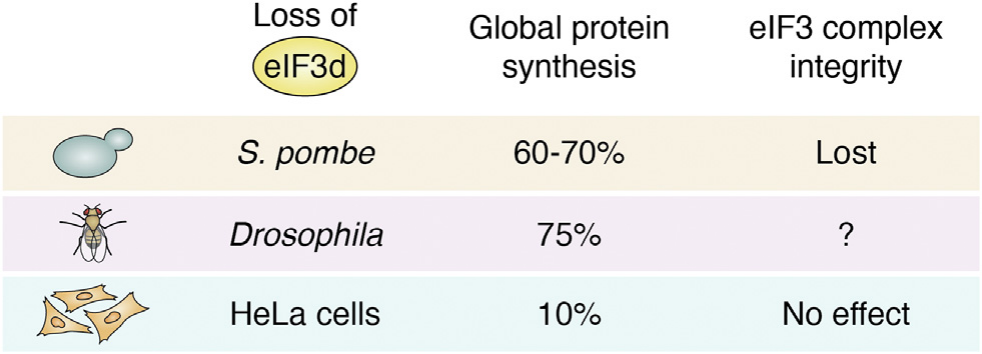
**The differences and similarities in eIF3d regulation of global protein synthesis and eIF3 complex integrity in yeast, fruit fly, and human cells.** eIF3, eukaryotic initiation factor 3.

In *Drosophila*, eIF3d depletion has been shown to be critical for derepressing the expression of the limiting dosage compensation complex subunit gene *msl-2* (30). Translation of the *msl-2* mRNA is inhibited by binding of a repressor complex consisting of heterogeneous ribonucleoprotein 48, sex-lethal (SXL), and upstream of N-ras to the 5′-UTR of the *msl-2* mRNA. It was found that eIF3d, but not other eIF3 subunits, interacts directly with heterogeneous ribonucleoprotein 48 using coimmunoprecipitation and mass spectrometry analyses and that only *eIF3d* knockdown reduced the inhibitory effect of the repressor complex on *msl-2* mRNA translation. In addition, it was found that eIF3d depletion inhibited the translation of *msl-2* mRNA in the absence of SXL. However, in the presence of SXL, eIF3d knockdown increased *msl-2* mRNA translation, likely by derepressing SXL inhibition. Thus, it was thought that the repressor complex binds to eIF3d and prevents eIF3d-mediated translation initiation of *msl-2* mRNA. Interestingly, *eIF3d* depletion did not cause major defects in global mRNA translation (20), which contrasts with studies in mammalian cells but is consistent with studies in *S. pombe* (Fig. 4). It was also shown using *in vitro* translation and pull-down assays that eIF3d was recruited to the 5′-UTR of *msl-2* mRNA in a fashion that correlates with cap-independent repression (30). However, the detailed mechanism is still unclear on how eIF3d works in the repressor complex–mediated suppression of *msl-2* mRNA translation. If eIF3d was required for initiation of the *msl-2* mRNA translation, its depletion would be expected to reduce translation of *msl-2 5′*-UTR-driven reporter or *msl-2* mRNA irrespective of SXL presence. However, *eIF3d* knockdown alleviated the repressor-mediated suppression in the presence of SXL, indicating that SXL requires eIF3d to repress *msl-2* mRNA translation. These observations are confusing regarding the relationship between eIF3d and the repressor complex and their functions in coordinating *msl-2* mRNA translation or its repression. Nevertheless, the aforementioned findings of eIF3d involvement in regulating *msl-2* mRNA translation are intriguing and open a possibility that there may be other mRNAs that are under similar translational control.

### Stress adaptation

During metabolic stress, cells undergo translation shutoff mediated by inactivation of eIF4E (42, 43). However, how mRNAs encoding stress response proteins escape translation shutoff remains unclear. The observation that eIF3d-mediated cap-dependent translation occurs upon mTOR inhibition and loss of eIF4E function as discussed above suggests that eIF3d may promote synthesis of proteins important for stress response. Indeed, recent evidence indicates that eIF3d is activated in response to metabolic stress, and it could mediate stress-induced mRNA translation in human cells to synthesize stress response proteins (43).

**Figure 5:**
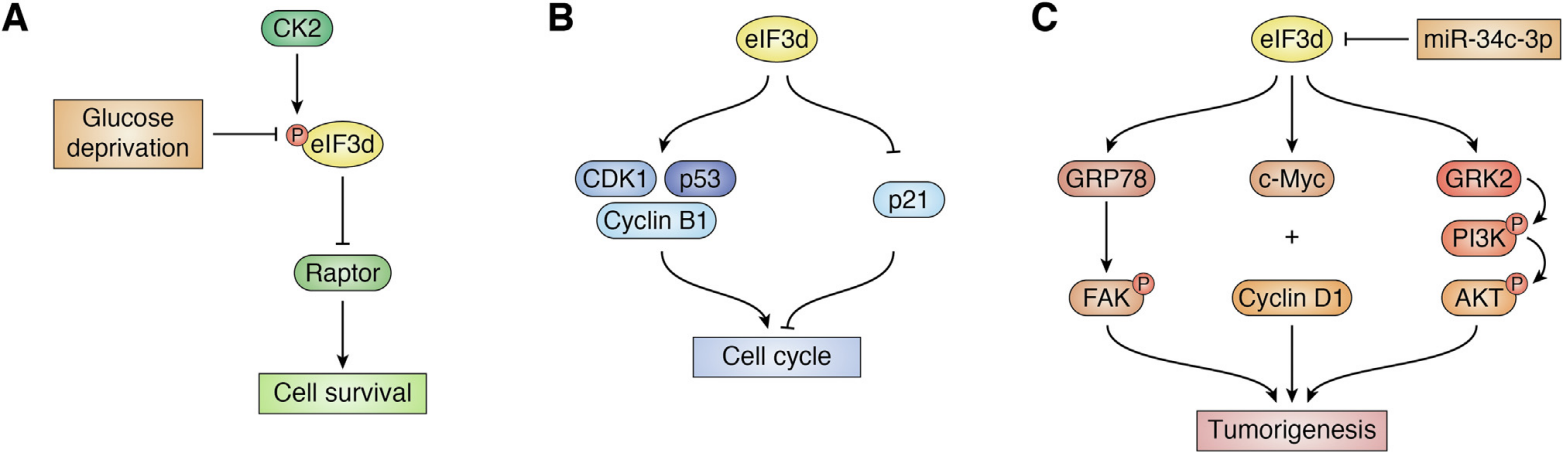
**Signaling pathways of eIF3d**. Glucose deprivation adaptation (*A*), cell cycle control (*B*), and tumorigenesis (*C*). CDK1, cyclin-dependent kinase 1; CK2, casein kinase 2; eIF3, eukaryotic initiation factor 3; FAK, focal adhesion kinase; GRK2, G protein–coupled receptor kinase 2; GRP78, glucose-regulated protein 78.

It was recently found that, during chronic glucose deprivation stress, eIF3d-driven *c-Jun* mRNA translation and eIF3d binding to the cap of *c-Jun* mRNA were both increased as determined using Western blot analysis of c-Jun and RT–PCR analysis of RNAs bound to the eIF3d CBD following HIV-1 protease (PR) digestion (43) (Fig. 5*A*). Because phosphorylation is a common mechanism in regulating eIF function during stress (44, 45, 46, 47, 48, 49), the glucose-dependent phosphorylation of eIF3d was examined. Using ^32^P-orthophosphate labeling and mass spectrometry approaches, the phosphorylation sites in eIF3d were mapped to Ser^528^ and Ser^529^, and casein kinase 2 was identified as the responsible enzyme to phosphorylate these Ser residues. Mutational analyses of these phosphorylation sites further revealed that unphosphorylated eIF3d facilitated selective translation of mRNAs including *Raptor* mRNA, and cell survival, during glucose deprivation. These findings suggest that eIF3d may drive noncanonical selective translation of mRNAs in cell stress response and survival. However, whether eIF3d-mediated selective mRNA translation is only in response to specific stimuli and if there are other regulators for eIF3d function in selective mRNA translation remain to be determined.

eIF3d has also been suggested to contribute to adaptation of endoplasmic reticulum (ER) stress. ER stress results from saturation of its capacity for synthesis and folding of nascent proteins. During acute/transient ER stress, eIF2B guanine nucleotide exchange factor is activated in response to this stress to regulate protein synthesis (50). However, during chronic ER stress, mRNA translation is independent of eIF2B guanine nucleotide exchange factor, and remodeling of the translation initiation is also independent of eIF4F (51). Recently, it was found using UV crosslinking and SDS-PAGE that eIF3 engages in translational activation of a subset of mRNAs during chronic ER stress (51). Moreover, by depleting selected eIF3 subunits (eIF3a, eIF3c, eIF3d, and eIF3g) and monitoring translational recovery during ER stress, it was found that eIF3d promoted translation of selected mRNAs such as *BiP* and *ATF4* and their association with 40S ribosomes, leading to translational recovery during chronic ER stress in an eIF4F-independent manner (51). However, it remains to be determined how eIF3d contributes to the recovery in translations of these specific mRNAs during ER stress.

## eIF3d in pathology and disease

Accumulating evidence indicates that eIF3d dysregulation contributes to tumorigenesis, cancer drug resistance, pre-eclampsia, and viral infection. In the following sections, we will review recent studies to understand the underlying mechanisms of eIF3d involvement in these four human pathological conditions.

### Cancer cell proliferation and cell cycle progression

Morphological abnormalities and unlimited proliferation are common hallmarks of tumor cells (52, 53). Examination of human cancer tissues has revealed that eIF3d expression is upregulated in many cancers including cancers of breast (54), prostate (55), ovary (56), gallbladder (21), stomach (57), bladder (58), colon (59), lung (60), and cervix (61) as well as in glioma (62), renal cell carcinoma (63), and chronic hepatitis C–associated hepatocellular carcinoma (64). These findings suggest that eIF3d may associate with tumorigenesis or cancer cell proliferation. Indeed, in a large-scale loss-of-function screening study using a genome-wide siRNA library, it was found that eIF3d might be involved in regulating mesothelioma cell proliferation and apoptosis (50). Furthermore, *eIF3d* silencing using shRNA significantly reduced proliferation of human melanoma cells as determined using methylthiazoletetrazolium (MTT) and colony formation assays (65).

While it is unknown how eIF3d regulates cell proliferation and survival, it has been suggested that eIF3d regulates cell cycle progression, which contributes to the proliferation and survival (Table 1). The involvement of eIF3d in cell cycle regulation has been reported in different cancer cell types (21, 54, 55, 56, 62, 63, 65, 66, 67, 68). For example, in the *eIF3d* knockdown study of melanoma cells, it was found that the loss of cell viability might be due to *eIF3d* knockdown–induced cell cycle arrest at G2/M phase (65). Similar findings have been described in studies using human renal cell carcinoma, acute myeloid leukemia, and prostate, ovary, colon, and non–small cell lung cancer cells (55, 56, 63, 66, 67, 68). However, it has also been observed that *eIF3d* knockdown using siRNA or shRNA expressed from lentiviral vector reduced cell proliferation, potentially because of arrest at G0/G1 phase with reduction of cells at S or G2/M phase in glioma and gallbladder and breast cancer cells (21, 54, 62). Although the cause for the discrepancy in these studies is unknown, these findings suggest that eIF3d may regulate cell proliferation by regulating cell cycle progression but in a cell line or cancer type–dependent manner (Table 1). It is also noteworthy that the molecular mechanisms by which eIF3d regulates cell cycle progression have not yet been investigated although eIF3d may translationally regulate the synthesis of CDK1, cyclin A, cyclin B1, p21, and p53, which are known to control cell cycle progression and, thus, may mediate eIF3d regulation of cell cycle (21, 65, 69) (Fig. 5*B*).

**Table 1:** eIF3d knockdown induced cell cycle distribution in human cancer cells

Cancer cell types	G0/G1 phase	S phase	G2/M phase	References
Breast cancer	Increased		Decreased	(54)
Colon cancer	Decreased	Decreased	Increased	(66)
Gallbladder cancer	Increased	Decreased	Decreased	(21)
Glioma	Increased	Decreased		(62)
Leukemia		Decreased	Increased	(68)
Non–small cell lung cancer	Decreased		Increased	(67)
Melanoma	Decreased	Decreased	Increased	(65)
Ovarian cancer		Decreased	Increased	(56)
Prostate cancer	Decreased		Increased	(55)
Renal cell carcinoma	Decreased		Increased	(63)

Although the mechanism of eIF3d involvement in cancer cell proliferation has not been elucidated, there is evidence that eIF3d may be involved in several signaling pathways that could affect cancer-related processes (Fig. 5*C*). First, ectopic overexpression of eIF3d significantly promoted the proliferation, migration, and stem cell–like properties of cervical cancer cells *via* activating the glucose-regulated protein 78 (GRP78)–focal adhesion kinase pathway (61). Similar findings have been made with ectopic eIF3d overexpression in colon cancer cells in which increased cell proliferation was observed as determined using colony formation, MTT, and soft-agar assays (59). eIF3d may regulate c-Myc and cyclin D1 expression, which in turn promotes colon cancer cell proliferation (59). It was also found that the 3′-UTR sequence of *eIF3d* mRNA contains a target site of miR-34c-3p (Fig. 2*A*) and that miR-34c-3p may regulate colon tumorigenesis *via* eIF3d as determined using MTT and soft-agar assays (59).

eIF3d may also promote prostate cancer cell proliferation as an m^6^A reader, which could be reversed by knocking down the expression of m^6^A eraser, ALKBH5 (70). Among a group of m^6^A readers identified in primary prostate cancer cells using multiomic approach, eIF3d was found to be a good prognosis factor. The findings that eIF3d promotes prostate cancer cell proliferation but predicts better prognosis are intriguing but are not entirely unexpected. Previously, it has been shown that eIF3a has similar functions in promoting tumorigenesis and cancer cell proliferation but contributes to better prognosis by suppressing synthesis of DNA damage repair proteins and promoting cancer cell response to DNA-damaging treatments (71, 72, 73, 74, 75, 76).

A yeast two-hybrid assay with eIF3d as a bait led to identification of G protein–coupled receptor kinase 2 (GRK2) as an eIF3d-interacting partner (21). Moreover, reciprocal coimmunoprecipitation and Western blot analysis revealed that eIF3d physically interacts with GRK2. *eIF3d* knockdown using shRNA in a lentiviral vector led to a decrease in GRK2 protein but not *GRK2* mRNA. Consistently, *eIF3d* overexpression increased the level of GRK2 protein. Furthermore, immunohistochemical staining showed that GRK2 expression correlated with eIF3d in human gallbladder cancer specimens, consistent with the possible eIF3d regulation of *GRK2* expression. Interestingly, eIF3d may regulate the stability of GRK2, not its synthesis, suggesting that eIF3d may have an additional noncanonical function. Domain mapping analyses showed that the carboxyl terminal amino acid residues 309 to 548 in eIF3d are responsible for GRK2 binding and required for increasing GRK2 protein stability by blocking ubiquitin–proteasome-mediated degradation of GRK2 (21). *GRK2* overexpression rescued the *eIF3d* knockdown–induced reduction in cell proliferation, colony formation, migration capacity, and Akt phosphorylation (Ser^473^) (21). Thus, it is possible that GRK2 may mediate eIF3d function in oncogenesis and PI3K/AKT signaling and that eIF3d may regulate the stability of GRK2 protein.

Although the aforementioned signaling pathways are potentially under eIF3d regulation, it is unclear if they contribute to and mediate the role of eIF3d in different cellular processes and in tumorigenesis. Future systematic investigations including rescue studies are expected to elucidate the mechanism of eIF3d action and the missing link between eIF3d and other cellular signaling pathways in different types of human cancers.

### Cancer cell drug resistance

eIF3 subunits have key roles in cancer drug and radiation resistance (76, 77, 78). It has been shown using immunohistochemical staining and Kaplan–Meier survival analysis that high eIF3d expression is tightly associated with worse prognoses in patients with gastric, gallbladder, lung, and liver cancers (21, 57, 60, 79), whereas eIF3d expression in prostate cancer associates with better prognosis (70). Although it is unknown if the discrepancy between these studies is due to different cancer types, the fact that eIF3d associates with prognosis, better or worse, suggests that it may play a role in therapeutic response or in disease aggressiveness or progression. However, limited studies on the role of eIF3d in cellular response to anticancer drugs have been performed. In metastatic gastric cancer patients with acquired resistance against cisplatin and fluorouracil combination, eIF3d was upregulated and may have potential value in predicting survival (80). Whole transcriptome RNA-Seq in combination with real-time quantitative RT–PCR helped identify a fusion transcript *MYH9-eIF3d* with higher expression in the docetaxel-resistant DU145 cells (81).

Consistent with the aforementioned findings, eIF3d protein level is significantly upregulated in sunitinib-resistant renal cell carcinoma cell lines and tissues (82). *eIF3d* knockdown with shRNA reduced, whereas *eIF3d* overexpression increased sunitinib resistance of the renal cell carcinoma cell lines (82). Mechanistically, eIF3d promoted the sunitinib resistance of renal cell carcinoma partially by blocking the ubiquitin-mediated proteasome degradation of GRP78. *GRP78* overexpression induced sunitinib resistance and restored the sunitinib resistance reduced by *eIF3d* knockdown *via* triggering the unfolded protein response signaling pathway, and conversely, *GRP78* knockdown inhibited colony formation capacity. These interesting findings are consistent with another study that showed that eIF3d regulates the stability of GRK2 (21). Whether eIF3d regulates GRP78 stability by directly binding to GRP78 as it does to GRK2 as discussed above remains to be determined.

### Viral infection

It has been reported that mammalian eIF3 and its subunits could mediate the viral infection process. For example, *eIF3m* silencing by siRNA reduced Herpes simplex virus plaque formation *via* inhibiting the translation of the immediate early viral proteins (83). eIF3l was found to interact with the NS5 viral protein (84). NS5 is essential for viral replication, and eIF3l overexpression facilitates global translation and shows a slight effect on the replication of yellow fever virus (84). A recent transcriptome analysis by capturing the ribonucleoprotein complex of coronaviruses showed that SARS-CoV-2 may recruit eIF3d to regulate cap-dependent translation initiation of SARS-CoV-2 genomic RNA (25). *eIF3d* knockdown using siRNA led to a significant reduction in viral RNAs, suggesting that it may be a proviral candidate protein for SARS-CoV-2 (25). However, it remains to be determined how eIF3d recognizes and regulates translation of SARS-CoV-2 transcripts.

HIV relies on host cellular machinery to replicate and attack the immune system, leading to AIDS. Many host proteins are targeted by viral proteins for interaction. Using affinity tagging and purification mass spectrometry in human embryonic kidney 293 and Jurkat cells, Jager *et al.* (85) revealed 497 HIV-human protein–protein interactions. Of these proteins, 12 of 13 eIF3 subunits (all except eIF3j) were found to interact with viral proteins. However, only eIF3d was found to be cleaved by recombinant HIV-1 PR protease following cotransfection of FLAG-tagged eIF3d and active HIV-1 PR protease in human embryonic kidney 293 cells. In addition, purified human eIF3 complex incubated with recombinant HIV-1 PR *in vitro* confirmed that eIF3d could be cleaved to a 60-kDa protein product. However, whether endogenous eIF3d is cleaved by HIV protease during HIV infection has yet to be shown and should be investigated. Nevertheless, amino-terminal sequencing of the cleaved eIF3d product showed that the cleavage occurs between Met^114^ and Leu^115^, which is located in the RNA-binding domain of eIF3d (29) (Fig. 2). In another study, HIV infectivity was measured using a pseudotyped virus expressing vesicular stomatitis virus glycoprotein (HIV–VSV-G), which only allows for a single round of replication, to determine the role of eIF3 subunits in HIV infection (85). Only *eIF3d* knockdown led to an increase in HIV infectivity, suggesting that eIF3d but not other eIF3 subunits may suppress early HIV infection/replication, possibly by binding to the viral RNA *via* its RNA-binding domain.

The mRNA level of eIF3d, among eIF3 subunits tested including eIF3b, c, d, k, l, and m, was found to markedly decrease in rapid progressors compared with chronic progressors after HIV infection, and *eIF3d* mRNA levels were inversely correlated with HIV progression (86). In this study, five different cell subsets (CD4+ and CD8+ T cells, NK and B cells, and monocytes) of peripheral blood mononuclear cells from healthy controls were analyzed by flow cytometry. CD8+ T cells had the highest expression level of eIF3d. Thus, it is possible that eIF3d expression in CD8+ T cells may affect these cells in disease progression.

*eIF3d* knockdown using siRNA suppressed proliferation and interferon-γ secretion and effectively induced apoptosis of CD8+ T cells as analyzed using flow cytometry (86). RNA-Seq and network analyses combined with knockdown experiments revealed that SOCS-7 mediated eIF3d regulation of CD8+ T-cell survival. Although decreased eIF3d expression inhibits CD8+ T-cell proliferation and promotes apoptosis, it is unknown if eIF3d affects HIV disease progression by regulating viral replication in primary T cells. It is also unknown how SOCS-7 mediates eIF3d function in HIV infection and disease progression. These authors also evaluated HIV replication using a pNL4-3 pseudo-typed virus in Jurkat and CD4+ T cells and found that replication was enhanced when eIF3d was reduced by siRNA (86).

**Figure 6:**
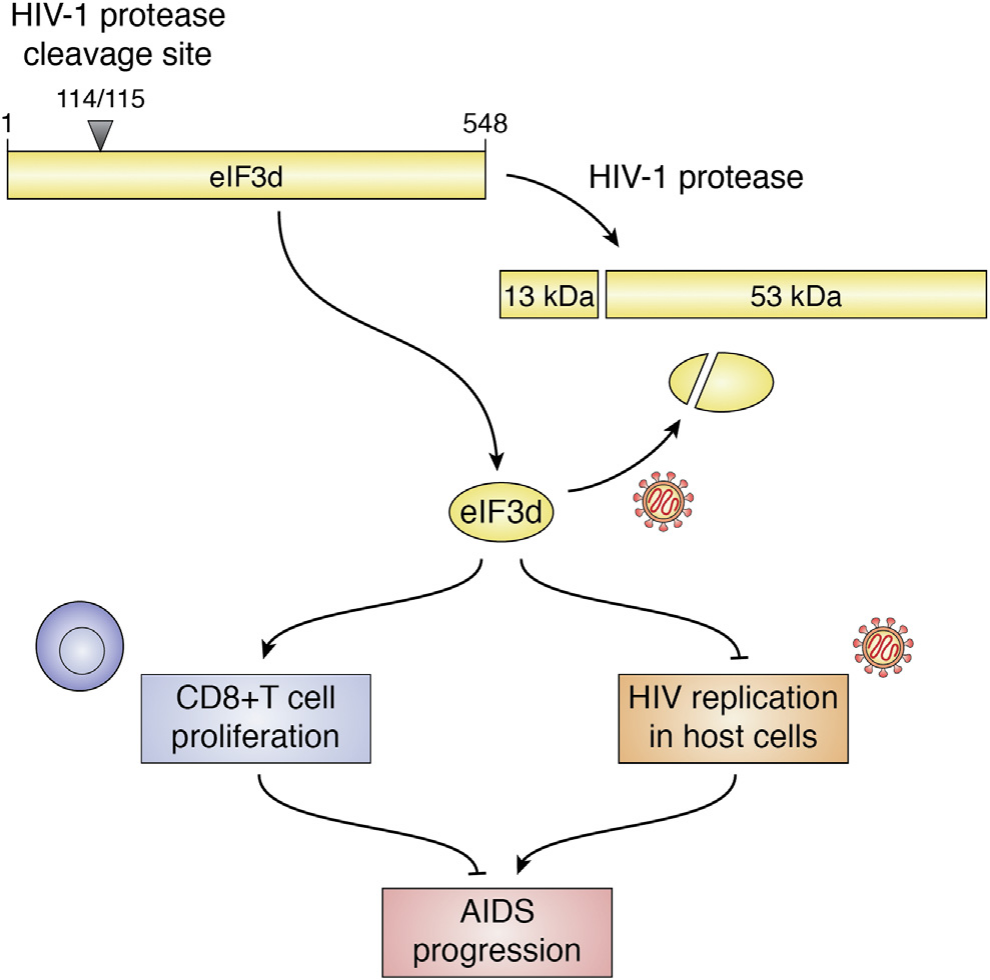
**Schematic model of eIF3d in HIV infection and AIDS progression by promoting CD8+ T-cell proliferation and inhibiting HIV replication in host primary T cells.** eIF3d cleavage by HIV-1 protease in host cells helps HIV defend against eIF3d suppression. eIF3d, eukaryotic initiation factor 3d.

Based on these findings concerning HIV infection, it is possible that eIF3d in the host cells infected by HIV virus inhibits the infection or replication of the virus possibly by inhibiting translation of the viral RNAs, whereas the virus counteracts the inhibition by cleaving eIF3d (Fig. 6). These possibilities clearly need additional detailed research to be tested and validated. Interestingly, increased eIF3d expression in effector CD8+ T cells also helps limit HIV infection and disease progression by regulating CD8+ T-cell proliferation and survival and interferon-γ secretion *via* SOCS-7 (Fig. 6). Although greater understanding of the mechanisms of eIF3d function in viral infection and in immunity is needed, eIF3d may be a potential prognosis marker and therapeutic target for AIDS patient.

In a recent study, it was found that the eIF3d protein level increased in response to human cytomegalovirus (HCMV) infection, whereas its mRNA level remained unchanged (87). Interestingly, silencing *eIF3d* expression using siRNA inhibited HCMV reproduction and reduced polyribosome abundance and virus protein accumulation. However, eIF3d depletion did not interfere with viral DNA synthesis. These findings suggest that eIF3d may regulate HCMV gene expression, but not its DNA replication, which leads to favorable viral replication. Moreover, the increased eIF3d expression during HCMV infection remodeled the global host mRNA translational landscape and switched the cap-dependent translation from an eIF4F- to an eIF3d-dependent mechanism to support productive viral replication. These important findings suggest that eIF3d could be developed as a target to inhibit HCMV infection.

### Pre-eclampsia

Pre-eclampsia is a complication of pregnancy and recognized as one reason for substantial neonatal and maternal morbidity and mortality. The relationship between eIF3d and preeclampsia was first identified in 2021 (88) in a study analyzing pre-eclampsia-related microarray datasets from the Gene Expression Omnibus database, which showed that eIF3d might facilitate pre-eclampsia progression. Indeed, it was found that *eIF3d* overexpression negatively impacted proliferation, invasion, migration, and angiogenesis of human trophoblast cell line HTR-8/SVneo as determined using MTT, EdU, transwell, wound healing, and tube formation assays (88). It is noteworthy that poor trophoblast invasion and excessive trophoblast apoptosis are thought to cause pre-eclampsia (89, 90, 91). It has also been found that *eIF3d* overexpression decreased the expression of proliferation, metastasis, and angiogenesis-related markers including cyclin A, PCAN, CDK1, N-cadherin, vimentin, VEGFA, and VEGFR2 (69, 92, 93). Consistent with these findings, *eIF3d* knockdown using siRNA suppressed pre-eclampsia progression, and the suppression is thought to be due to activation of the mitogen-activated protein kinase (MAPK)–ERK1/2 pathway by *eIF3d* knockdown (88). These findings suggest that high eIF3d may promote pre-eclampsia progression by negatively regulating the MAPK–ERK1/2 pathway. Whether eIF3d regulation of cyclin A, PCAN, CDK1, and phosphorylation of MAPK–ERK1/2 is due to eIF3d modulation in translation of specific mRNAs remains to be determined.

## Conclusion and perspectives

The function of eIF3d in maintaining the integrity of the eIF3 complex and in global translation initiation is controversial and debatable depending on the model system used for each study. While eIF3d is essential to maintain eIF3 complex integrity in fission yeast, its deletion and, thus, loss of the eIF3 complex resulted in only partial loss in global protein synthesis. Consistent with the findings using fission yeast, *eIF3d* depletion in *Drosophila* also had little impact on global protein synthesis while impacting the synthesis of specific proteins. In contrast, loss of mammalian eIF3d had little impact on the eIF3 complex integrity. However, eIF3d loss is detrimental to global protein synthesis in human cells. While further studies are clearly needed to delineate the causes for these differences, these findings suggest that eIF3d may have other noncanonical functions to control translational levels of specific subsets of mRNAs in different model systems. These findings also challenge the prevailing concept that the intact eIF3 complex is essential in global translation initiation.

The findings that eIF3d directly regulates specific mRNA translation by binding to the 5′-cap structure or 5′-UTRs of these mRNAs or interacting with other proteins such as DAP5 are very intriguing. It is unclear currently if the noncanonical function of eIF3d in cap-dependent or cap-independent translation initiation requires the eIF3 complex or other subunits of eIF3 (Fig. 3, *B* and *C*). This noncanonical function in translational control may contribute to regulation of stress adaptation, cell cycle progression, virus infection, and disease onset and progression. However, it is noteworthy that manipulating the level of an eIF3 subunit genetically may affect the integrity, stability, or structure of the eIF3 complex that may in turn affect eIF3 function in binding and scanning process during the initiation step. Although *eIF3d* knockdown had little impact on the eIF3 complex integrity in mammalian cells, it is yet unknown if eIF3d downregulation alters eIF3 complex structure or conformation. Furthermore, loss of eIF3d may cause the rearrangement of other subunits in the eIF3 complex even if the complex does not disintegrate. Similarly, it is unknown whether *eIF3d* overexpression results in more eIF3 complex if eIF3d is a limiting factor or results in excess eIF3d that exists and functions alone independent of the eIF3 complex. Clearly, future studies are necessary to address these challenging questions. Obtaining structural information of the eIF3 complex with or without eIF3d or any other subunit that does not influence eIF3 complex integrity *via* X-ray crystallography, cryo-EM, or Bio-SAXS in combination with reconstituted *in vitro* translation systems for binding and translation of noncanonical mRNAs with or without eIF3 or eIF3d should help address some of these questions.

Furthermore, eIF3d is an understudied protein compared with other eIF3 subunits such as eIF3a and eIF3b, and many additional questions remain to be answered. First, there is little understanding of how eIF3d expression is regulated and of the signals involved in upregulating eIF3d expression that leads to pathological alterations. Second, no *in vivo* animal models have been developed and used to demonstrate the role of eIF3d in disease progression and protein synthesis. Third, the findings that eIF3d binds to and regulates the stability of GRK2 and GRP78 are very exciting, suggesting that eIF3d may have noncanonical function in addition to translational regulation of specific mRNAs. Whether eIF3d regulates other proteins by controlling protein stability needs to be investigated. Finally, more efforts are needed to explore the possibility of establishing eIF3d as a biomarker and potential therapeutic target for cancer and AIDS. The finding of eIF3d in regulating CD8+ T-cell survival is important, and further studies are needed to determine how eIF3d functions in immunity and how eIF3d could possibly be developed as a target for immunotherapy and prevent viral infection.

## Data availability

All data are available in the article. No experimental raw data were generated or used for this article.

## Conflict of interest

The authors declare that they have no conflicts of interest with the contents of this article.
